# Cytoprotective Effect of *Lactobacillus crispatus* CTV-05 against Uropathogenic *E. coli*

**DOI:** 10.3390/pathogens5010027

**Published:** 2016-03-08

**Authors:** Daniel S. C. Butler, Aurelio Silvestroni, Ann E. Stapleton

**Affiliations:** 1 Department of Microbiology, Immunology and Glycobiology, Institute of Laboratory Medicine, Lund University, 221 00 Lund, Sweden; daniel.butler@med.lu.se; 2 Department of Medicine, Division of Allergy and Infectious Diseases, University of Washington, Seattle, WA 98195-5852, USA; aurels@uw.edu

**Keywords:** urinary tract infection, *Lactobacillus crispatus*, innate immunity, probiotics

## Abstract

The vaginal flora consists of a subset of different lactic acid producing bacteria, typically creating a hostile environment for infecting pathogens. However, the flora can easily be disrupted, creating a favorable milieu for uropathogenic *Escherichia coli* (UPEC), making it possible to further infect the urinary system via the urethra. Probiotic use of different *lactobacilli* to restore the normal flora of the vagina has been proposed as a potential prophylactic treatment against urinary tract infections. This project evaluated the protective- and anti-inflammatory roles of the probiotic *Lactobacillus crispatus* strain CTV-05 in an *in vitro* system. The inflammatory response and the cytotoxic effect were studied by Enzyme-linked immunosorbent assays and by trypan blue exclusion of cells inoculated with *L. crispatus* CTV-05 and comparing it to non-infected controls and UPEC infected cells. *L. crispatus* CTV-05 showed no cytotoxicity to vaginal epithelial cells compared to non-infected controls and provided significant protection against UPEC infection (*p* < 0.05). Further more, *L. crispatus* CTV-05 did not create a pro-inflammatory response *in vitro*, with no significant increase of IL-1β or IL-6. These results demonstrate the protective effect of using *L. crispatus* CTV-05 as a probiotic treatment to reduce the risk of recurrent urinary tract infections.

## 1. Introduction

Recurrent acute uncomplicated cystitis (AUC) is a common infection, most often caused by *Escherichia coli* strains known as uropathogenic *E. coli* (UPEC) originating in the rectal microbiota. Women are far more affected than men. Half of the female population in the US will have at least one episode of acute cystitis during their lifespan, and around a third of these women will develop recurrent UTIs, resulting in increased morbidity and health care expenditures [1,2] A strong link between vaginal health and UTIs has been described and may serve as a basis for the skewed gender distribution of AUC [3,4,5]. The healthy vagina is colonized by different lactic acid producing bacteria (LAB), most commonly strains of *Lactobacillus crispatus*, *Lactobacillus jensenii* and *Lactobacillus iners* [6,7,8]. The vaginal flora is easily disrupted; several studies have indicated that frequent sexual activity [9], the use of spermicide contraceptives [10], and certain hygiene regimens [11] allow different pathogens to colonize vaginal epithelial cells (VECs) and establish a potential reservoir for future UTIs. After an episode of AUC, the healthy, protective vaginal microbiota may take weeks to re-establish and an increased risk of new vaginal colonization accompanies this period, increasing the risk for recurrent UTI [12]. This reservoir of bacteria may further invade the urethra, facilitating the bacterial spread and infection of the urothelium of the bladder or the renal pelvis, thus precipitating clinical disease [13,14].

Various normally resident *Lactobacillus* spp. have been postulated to protect the vagina from invading pathogens by means of mechanisms including the acidification of the vagina creating an unfavorable milieu for invading pathogens. In addition, *Lactobacillus* adhesion to the vaginal epithelium and the resulting competitive exclusion of adhering UPEC strains has been proposed as a mechanism for both resident and probiotic *Lactobacilli* to impair UPEC colonization and infection. Several successful studies have been performed using *Lactobacilli* to re-establish the normal vaginal microbiota, establishing the efficacy of prophylactic lactobacilli in replenishing the vaginal flora. In addition, this approach might avoid the negative effects of antibiotics, some of which alter the vaginal microbiota [15,16,17,18].

The severity of UTI reflects the magnitude and functionality of the innate immune response to infection. Adhering UPEC bacteria stimulate a cytokine response in uroepithelial and vaginal cells [19,20]. IL-1β and IL-6 concentrations are elevated in the urine of patients with UTI following infection. In acute pyelonephritis, blood cytokine levels are augmented and play a role in the generation of fever and the acute phase response [21].

The use of probiotics needs to be further explored as a potential prophylactic and therapeutic measure against uropathogens with increasing resistance to antibiotics. In this study we used an immortalized primary cultured vaginal epithelial cell (VEC) system [22] as an *in vitro* model of the protective effects of the probiotic *L. crispatus* strain CTV-05. We evaluated the effects on UPEC cytotoxicity, adherence, and pro-inflammatory response activation, quantifying pro- and anti-inflammatory cytokines regulated by UPEC infection.

## 2. Results and Discussion

### 2.1. Results

#### 2.1.1. *L*. *crispatus* CTV-05 Reduces the Toxicity of UPEC Strains to Vaginal Epithelial Cells

**Figure 1:**
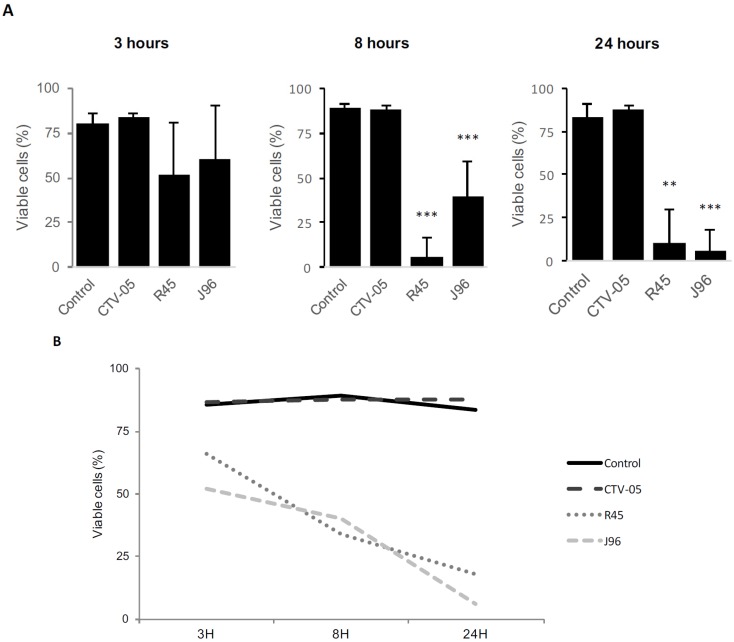
*L. crispatus* CTV-05 is non-toxic to vaginal epithelial cells compared to non-infected control cells and UPEC infected cells. Vaginal epithelial cells were inoculated with the probiotic *L. crispatus* strain CTV-05 (10^3^ cfu/mL), the cystitis isolate *E*. *coli* R45 (10^8^ cfu/mL) or the pyelonephritis strain *E*. *coli* J96 (10^3^ cfu/mL) for 3, 8 or 24 h. (**A**) Graphs illustrate the average percentage of viable cells after infection for the three different time points (3 h to the right, 8 h in the middle, and 24 h to the left) using trypan blue exclusion. Statistical significance was calculated using paired *t*-test. ** *p* < 0.01; *** *p* < 0.001; (**B**) Line graph illustrating the cytotoxic effect of *L. crispatus* CTV-05, *E*. *coli* R45 and *E*. *coli* J96 compared to non-infected control cells over 24 h using trypan blue exclusion.

To exclude cytotoxicity of *L. crispatus* CTV-05, a cell viability assay was performed using VECs immortalized with the amphotropic retrovirus vector, *LXSN16E6E7* (gift from D. Galloway, Seattle, WA, USA) containing the E6 and E7 genes of HPV 16 with the neomycin resistance gene. The loss of cell viability after *in vitro* infection with *L. crispatus* CTV-05 was quantified by trypan blue exclusion compared to non-infected cells and bacterial numbers were determined by viable counts of the supernatants, 3, 8, and 24 h after incubation with the VECs. *L. crispatus* CTV-05 did not alter cell viability (10^8^ cfu/mL), mean viability 87.8% ± 5.1%, 24 h, *p* = 0.15 compared to non-stimulated VECs (Figure 1a,b).

In contrast, the UPEC strains *E*. *coli* R45 and *E*. *coli* J96 were cytotoxic for the VECs. This effect was dose-dependent for cells infected with 10^5^, 10^4^ and 10^3^ cfu/mL. To avoid excessive cytotoxicity, 10^3^ cfu/mL was selected for further studies. The average viability of non-stimulated VECs showed no significant difference between the three time-points analyzed.

**Figure 2:**
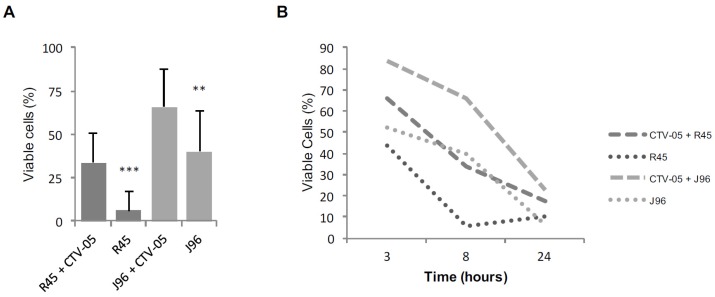
*L. crispatus* CTV-05 reduces the cytotoxicity of UPEC strains when pre-inoculating vaginal epithelial cells for 1 h prior to UPEC exposure. (**A**) The cytoprotective role of *L. crispatus* CTV-05 was studied by pre-inoculating cells with *L. crispatus* CTV-05 one hour prior to UPEC exposure for 8 h compared to cells only inoculated with the UPEC strains *E*. *coli* R45 (10^3^ cfu/mL) or *E*. *coli* J96 (10^3^ cfu/mL). Percentage of viable cells after eight hours was determined using trypan blue exclusion. Statistical significance was calculated using paired *t*-test. ** *p* < 0.01; *** *p* < 0.001; (**B**) Percentage of viable cells when comparing the means for three different time-points, 3, 8 and 24 h of cells pre-treated with *L. crispatus* CTV-05 for 1 h prior to UPEC infection compared to cells only infected with UPEC strains.

To address if *Lactobacilli* may protect against the cytotoxicity of the UPEC strains, VECs were pre-inoculated with 10^8^ cfu/mL of *L. crispatus* CTV-05 for 1 h prior to UPEC infection and cell death was measured by trypan blue exclusion, as described above. Pre-inoculation of *L. crispatus* CTV-05 yielded significant differences between cells exposed to *L. crispatus* CTV-05 before inoculation with *E*. *coli* J96 and VECs only infected with *E*. *coli* J96 (*p* < 0.001 after 8 h), The same effects were seen with *E*. *coli* R45, where pre-inoculation with *L. crispatus* CTV-05 for one hour yielded a significant difference compared to *E*. *coli* R45 stimulated cells (*p* < 0.001 after 8 h) (Figure 2a).

#### 2.1.2. *L. crispatus* CTV-05 Reduces the Adherence of UPEC to Vaginal Epithelial Cells

**Figure 3:**
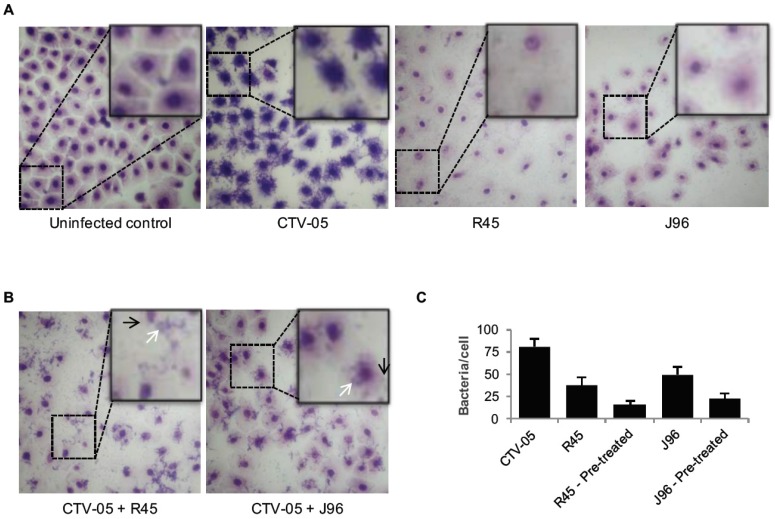
*L. crispatus* CTV-05 reduces the binding of UPEC strains. (**A**) Vaginal epithelial cells were inoculated with *L. crispatus* CTV-05, *E. coli* R45 or *E. coli* J96 for 1, or 24 h respectively and stained using a modified Giemsa stain to visualize the kinetics of adhering bacteria; (**B**) Vaginal epithelial cells pre-treated with *L. crispatus* CTV-05 for 1 h before inoculation using UPEC strains *E*. *coli* R45 or *E*. *coli* J96 to see if UPEC adherence was reduced when pre-treated. Black arrows indicate *L. crispatus* CTV-05 binding; white arrows indicate UPEC binding. (**C**) Number of bacteria per cell was determined by counting adhering bacteria to 10 cells and calculating the average adhering *L. crispatus* CTV-05, *E. coli* R45, *E. coli* R45 after 1 h of *L. crispatus* CTV-05 pre-treatment, *E. coli* J96 and *E. coli* J96 after 1 h of *L. crispatus* CTV-05 pre-treatment.

The kinetics of bacterial adherence was quantified by light microscopy after incubation of the VECs with bacteria for 24 h, fixing and staining using a modified Giemsa stain. *L. crispatus* CTV-05 (10^8^ cfu/mL) showed a mean adherence of 81 bacteria per cell. The UPEC strains also adhered efficiently, with a mean of 38 bacteria per cell for *E*. *coli* R45 (10^3^ cfu/mL) and 50 bacteria per cell for *E*. *coli* J96 (10^3^ cfu/mL). Pre-inoculation of the VECs with *L*. *crispatus* CTV-05 for 1 h reduced UPEC adherence to 16 and 23 bacteria per cell for *E*. *coli* R45 and *E*. *coli* J96, respectively (Figure 3b).

#### 2.1.3. *Lactobacillus crispatus* CTV-05 Products in Spent Broth Filtered Supernatant Inhibit(s) the Growth of UPEC

**Figure 4:**
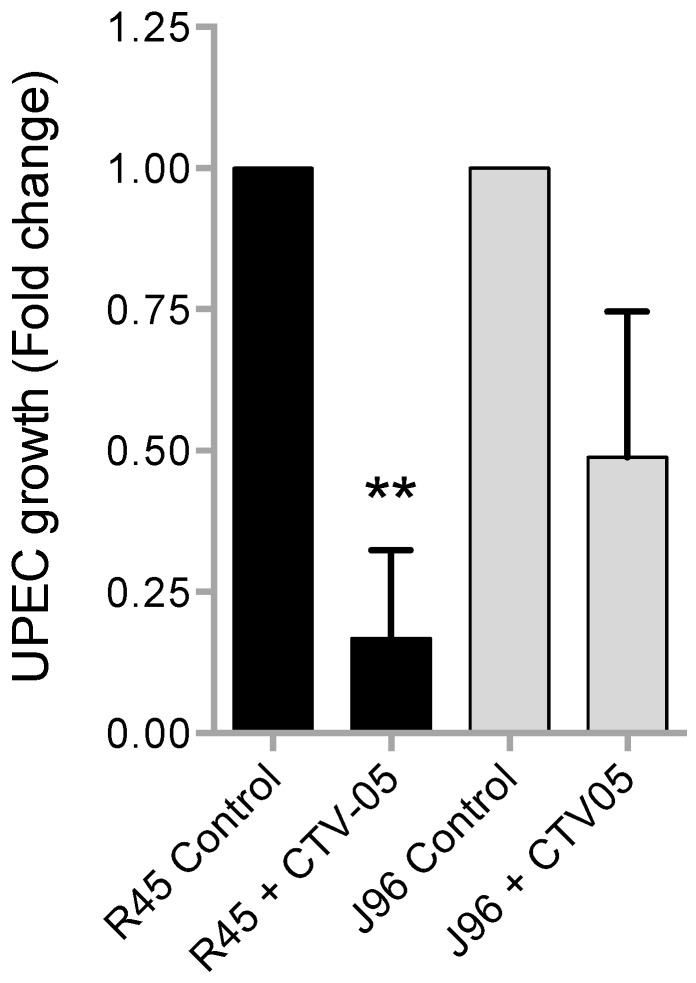
*L. crispatus CTV-05 products slow UPEC growth.* UPEC bacteria were grown in either control solutions (MRS/BHI-broth media) or an active solution (filtered *L. crispatus* CTV-05 supernatant) for 24 h before determination of bacteriostatic effect of *L. crispatus* CTV-05 products on UPEC growth. The graph shows the fold change of UPEC growth in an active solution as compared to control solution. (Means + SEM, Paired *t*-test, ** *p* < 0.01)

To investigate whether the probiotic *L. crispatus* strain CTV-05 had effects other than steric hindrance, a bacterial growth inhibition assay (modification of Osset *et al.* [23]) was performed by incubating UPEC strains with a sterile filtered supernatant from a 24 h culture of *L. crispatus* CTV-05 prior to measurement of UPEC growth. These results demonstrated that the filtered spent broth supernatant from *L. crispatus* strain CTV-05 inhibited the growth of UPEC. UPEC grown in the presence of *L. crispatus* CTV-05 supernatant yielded a lower percentage growth than the control counterpart. *E*. *coli* R45 grown in presence of *L. crispatus* CTV-05 supernatant yielded only 45% on average of the growth compared to the *E*. *coli* R45 control, *p* < 0.01. The same trend could be seen in *E*. *coli* J96 grown in the presence of *L. crispatus* CTV-05 supernatant compared to the control counterpart, 48% on average (Figure 4).

#### 2.1.4. *L. crispatus* CTV-05 Anti-Inflammatory Response in Vaginal Epithelial Cells

The innate immune response to *L. crispatus* CTV-05 was quantified after inoculation of the VECs for 8 h. There was no significant difference in any of the studied cytokines when comparing *L. crispatus* CTV-05 inoculated VECs (10^8^ cfu/mL) and uninfected VECs.

When infecting VECs with the different UPEC strains (10^3^ cfu/mL), the IL-1β concentration was significantly elevated compared to uninfected controls. VECs incubated with *E*. *coli* R45 showed a median concentration of 57.42 pg/mL (*p* < 0.05) and those incubated with *E*. *coli* J96 showed a median concentration of 59.52 pg/mL (*p* < 0.05), compared to uninfected VECs, which produced 2.49 pg/mL. There was no significant difference between UPEC infected VECs and uninfected VECs for IL-6 or IL-10 (Table 1).

To address if the *L. crispatus* CTV-05 strain inhibited the adherence of the UPEC strains used in this study (*E*. *coli* R45 and *E*. *coli* J96), VECs were pre-inoculated for 1 h with *L. crispatus* CTV-05 (10^8^ cfu/mL) before UPEC infection (10^3^ pg/mL). IL-1β secretion was decreased compared to UPEC infection alone. Pre-inoculating VECs with *L. crispatus* CTV-05 before *E*. *coli* J96 stimulation decreased IL-1β concentration significantly, 14.74 pg/mL compared to 59.52 pg/mL for *E*. *coli* J96 stimulated cells alone (*p* < 0.05) The same trend was seen in *E*. *coli* R45 stimulated cells; however, the difference did not reach statistical significance (35.10 pg/mL compared to 57.42 pg/mL when stimulated with *E*. *coli* R45 alone). There were no significant differences in either IL-6 or IL-10 concentrations when pre-inoculating cells with *L. crispatus* CTV-05 (Table 2).

#### 2.1.5. Clinical Data Suggest the Same Cytokine Expression Trend as *In Vitro* Data

To test whether trends seen in the *in vitro* data correlated with findings in clinical specimens, periutheral samples from women diagnosed with acute uncomplicated cystitis were analyzed and compared with samples from the same women two weeks following treatment of their urinary tract infection with fosfomycin.

**Figure 5:**
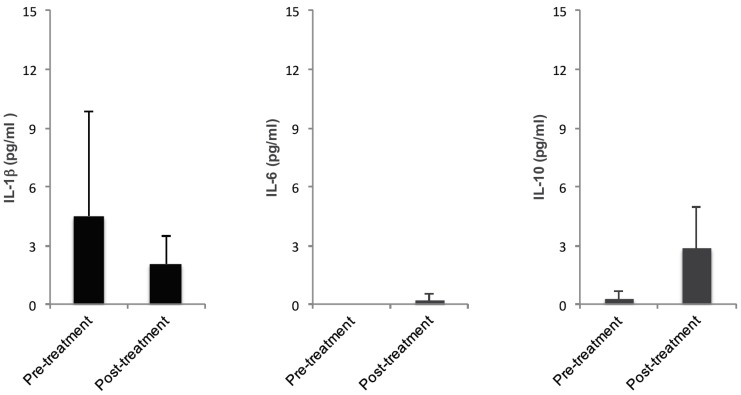
Cytokine response in women with AUC before and after fosfomycin treatment. Enzyme linked immunosorbent assays analyzing IL-1β, IL-6 and IL-10 concentration were performed on periutheral clinical samples from women diagnosed with AUC. Graph shows the average concentration of IL-1β, IL-6, and IL-10 before and two weeks post fosfomycin treatment.

IL-1β concentration was elevated pre-treatment compared to post-treatment (4.50 pg/mL *vs.* 2.09 pg/mL) but not significantly (*p* = 0.49). IL-6 concentration showed no increase pre- or post-treatment (0 pg/mL *vs.* 0.19 pg/mL), *p* = 0.34. IL-10 concentrations were lower pre-treatment than post treatment (0.26 pg/mL *vs.* 2.87 pg/mL) but was not significantly increased post-treatment (Figure 5).

### 2.2. Discussion

Studies have repeatedly demonstrated that vaginal colonization with UPEC often precedes ascension of the infecting bacterial strains to the urethra and bladder, resulting in AUC. The healthy vaginal microbiota in pre-menopausal women is characterized by various species of *Lactobacillus*, but this protective microbiota can be disrupted by exposures such as estrogen insufficiency or systemic antimicrobials [7,10], allowing UPECs to act as opportunists. Normalization of the disrupted vaginal flora using the probiotic strain *L*. *crispatus* CTV-05 has been tested in two prior trials as a preventative treatment for recurrent UTIs [24,25]. The present study investigated the cytoprotective effects of using the probiotic bacteria *L*. *crispatus* CTV-05 in an *in vitro* vaginal epithelial cell system as well as providing insight of how key inflammatory cytokines are regulated in VECs by exposure to *L*. *crispatus* CTV-05 as compared with UPEC infection.

In this work, the survival of VECs *in vitro* was clearly unaffected by exposure to *L*. *crispatus* CTV-05, as can be seen in Figure 1a-d. This is in marked contrast to the results of exposing nearly any cultured cell line to a pathogen, in which the mammalian cells are typically damaged and may detach. Thus, these data indicate this *in vitro* system is potentially useful in studying complex interactions between resident commensals and UPEC and other pathogens in the vagina. Although the mechanism of this cytoprotective effect is unknown, in studies of the gut epithelium showing similar effects, the apparent cytoprotective effect of lactobacilli is mediated by increased epithelial survival through regulation of cytoprotective heat shock proteins [26]. Another study found that lactobacilli may inhibit cytokine-induced apoptosis of the gut epithelium [27], promoting cell survival. Further study of the mechanism of this effect in the vaginal epithelium is merited.

VECs pre-incubated with *L*. *crispatus* CTV-05 prior to exposure to *E*. *coli* J96 had a significantly higher survival rate as compared to VECs infected with *E*. *coli* J96 alone after 8 h. However, pre-incubation with *L*. *crispatus* CTV-05 did not have the same protective effect against *E*. *coli* R45 effects after 24 h (Figure 2). Pre-incubation for 1 h with *L*. *crispatus* CTV-05 showed a limited protection after 24 h in respect to the non-infected cells (Figure 2). Presumably these differences are explained by the more rapid replication rates of *E*. *coli versus* lactobacilli as well as possible differences in the kinetics of *E*. *coli* production of cytotoxins such as hemolysin. Though both *E. coli* R45 and *E*. *coli* J96 produce hemolysin, testing the kinetics of hemolysin production or other known cytotoxins was beyond the scope of this study.

However, examining light microscopic images of the VECs after having been incubated with *L*. *crispatus* CTV-05 in a classic bacterial adherence assay, numerous *L*. *crispatus* CTV-05 bacteria were seen adhering to the VECs after 1 h of exposure (Figure 4). A potential explanation of these results could be the relative sizes of the different strains of UPECs. *E*. *coli* R45 is smaller than many UPEC and thus *L*. *crispatus* CTV-05 may be less effective at mediating steric hindrance of this UPEC. Other potential explanations of this phenomenon could be that the two different strains of *E*. *coli* express different virulence factors affecting the VECs directly and/or influencing the lactobacillus-mediated effects. Further investigation of this question would be aided by sequencing of the *E*. *coli* R45 genome and by virulence determinant expression assays.

The results of the growth inhibition assay suggested that the protective effect of *L*. *crispatus* CTV-05 was not merely due to steric hindrance of attachment. Although the growth of *E*. *coli* was not completely inhibited, the growth was reduced substantially as compared with the control. Previous research has found that some lactobacilli produce organic acids such as acetic acid and lactic acid, as well as H_2_O_2_ [28,29,30]. In vitro data suggest organic acids produced by lactic acid producing bacteria enter the cytoplasm of Gram-negative bacteria, lowering the intracellular pH and killing the bacteria [31]. Hydrogen peroxide is an oxidative agent able to kill bacteria, partly by damaging the bacterial DNA through oxidative stress [32,33]. *L*. *crispatus* CTV-05 produces both organic acids (Stapleton, University of Washington, unpublished work, 2010) and H_2_O_2_ [24], indicating that the probiotic produces a more hostile vaginal environment for invading pathogens. Other lactic acid producing bacteria are known to produce several bacteriocins and this could be another potential explanation of why UPEC is inhibited in this assay [34,35]. Another potential mechanism seen *in vivo* is the competition for nutrients between the commensal bacteria and the invading pathogens, thereby promoting slower growth of the invading pathogen, in this case different strains of UPEC.

The cytokine response in VECs further demonstrated the protective effect of *L*. *crispatus* CTV-05. The pro-inflammatory cytokine IL-1β was only slightly elevated compared to the non-infected controls but significantly lower than in both the UPEC infected VEC assays (Table 1). The anti-inflammatory cytokine IL-10 was elevated compared to the non-infected control, however UPEC infected cells, was even more elevated than the *L*. *crispatus* CTV-05 treated cells. Although this appears to be a paradoxical finding, given that this study represents the first characterization of this system, it is possible that IL-10 responses are actually pro-inflammatory in this model. Both *in vivo* and *in vitro* results from different studies have shown a diminished pro inflammatory response upon treatment with lactobacilli, citing immune-modulatory effects such as regulating the expression of TLR2 and TLR4 on the surface of HeLa cells [36]. There are also reports of soluble LAB molecules that reduce cytokine expression due to the down-regulation of NF- κB, which might be due to LAB inhibiting the Akt/PI3K signalling pathway [37].

Surprisingly, no elevation in IL-6 could be found upon UPEC stimulation of VECs although an increase in IL-6 levels have been demonstrated in other cell systems, including the urinary system, where binding of the bacteria causes a significant increase in IL-6 levels [38] The lack of IL-6 expression is supported partly by data from an *in vitro* study, showing that infected cells did not elevate IL-6 concentration compared to non-infected cells [39], but also from *in vivo* data from bacterial vaginosis patients [40,41]. These data suggest that UPEC binds and colonizes the vagina, but does not cause acute immune response as seen during bladder infections, perhaps because the vaginal mucosa is adapted to be continuously exposed to potential pathogens. Understanding this immunological response would be of significant importance not only for UTIs but also for other vaginal infections such as sexually transmitted diseases and bacterial vaginosis.

Activation of inflammatory cytokines by certain anaerobic Gram-positive bacteria was suggested by IL-1β and IL-6 measurements in mucosal secretions from patients with bacterial vaginosis [42,43]. We also measured cytokines in periurethral samples collected from healthy women and women with symptomatic uncomplicated cystitis accompanied by periurethral UPEC colonization. Comparing these data with the *in vitro* assay showed the same trend where IL-1β concentrations were higher in the untreated groups, however, IL-10 was increased when there were no UPEC present in the system (Figure 5). Although concentrations were lower *in vivo* than *in vitro*, the trends were the same indicating that the *in vitro* system is representative of the *in vivo* situation. It is also possible that instead of infecting cells as appears to occur in the *in vitro* system, UPEC only colonize the periurethral area and do not cause an inflammatory response. To further understand the cytokine response from clinical samples more samples need to be analyzed to get a representative picture of the whole system.

Further studies need to be performed to be able to understand the complete effect of using *L*. *crispatus* CTV-05 as a prophylactic treatment against recurrent UTIs and BV. To understand adhesion and the competitive effect against both UPEC and other vaginal pathogens is a key step in understanding the clinical value of using it as a preventative treatment against UTIs and re-establishing vaginal health. However, this study and prior *in vitro* studies suggest that using *L*. *crispatus* CTV-05 as a prophylactic treatment in patients who experience recurrent UTIs or bacterial vaginosis may have beneficial effects beyond merely reducing the rates of recurrence, supporting the concept of benefit to vaginal health by re-establishing the vaginal microbiota after antibiotic treatment.

## 3. Experimental Section

Fully keratinocyte characterized vaginal epithelial cells immortalized with human papilloma virus were grown in three 6-well plates with keratinocyte-serum free medium (K-SFM) until 90% confluent. Cells were inoculated with *L*. *crispatus* CTV-05 (10^8^ cfu/mL) grown in MRS broth for 48 h prior to inoculation, or the UPEC strains *E*. *coli* R45 and *E*. *coli* J96 (10^3^ cfu/mL) grown on MacConkey agar for 16 h in 37 °C prior to inoculation. *E. coli* R45 was isolated from a case of human cystitis and *E*. *coli* J96 (O4:K6) was isolated from human pyelonephritis patient. The infected cells were incubated for 3, 8, or 24 h and cells were harvested and percentage of viability was determined using trypan blue exclusion. Supernatant were harvested from the infected cells from the eight hour time point to perform IL-1β, IL-6 and IL-10 analysis using commercial enzyme linked immunosorbent assay (ELISA) kits from R & D systems (Minneapolis, MN, USA). Bacterial adhesion was studied using a modified Giemsa stain (DADE Behring, Newark, DE, USA). Cells were analyzed using Olympus BX50 microscope using 60× magnification and photographed with a cannon EOS 5D camera.

UPEC growth inhibition was studied by incubating UPEC strains *E*. *coli* R45 and *E*. *coli* J96 together with filtered supernatant from *L. crispatus* CTV-05*. E*. *coli* R45 and *E*. *coli* J96 were grown on blood agar for 24 h prior to the experiment and *L. crispatus* CTV-05 was grown in MRS-broth for 48 h. *L. crispatus* CTV-05 was then diluted to 10^6^ cfu/mL and filtered through a 0.22 um filter (Millipore Corporation, Billerica, MA, USA) and put into BHI broth in a 1:3 ratio. UPEC strains were diluted to a concentration of 10^7^ cfu/mL in BHI broth before adding the *L. crispatus* CTV-05 filtered supernatant, or a 1:3 ratio BHI-/MRS broth solution as a control, in a one part UPEC-, nine part supernatant/control-solution ratio before incubating for 24 h. UPEC were then plated on blood agar for 24 h before counting cfu/mL for the different conditions.

For *in vivo* cytokine measurements, cotton swabs received from clinical examination of women with symptomatic uncomplicated acute cystitis and periutheral UPEC colonization were used and compared to women following fosfomycin treatment. Patients recruited for the study had provided written informed consent under a University of Washington IRB approved protocol. Protected health data were de-identified and specimens were coded so that only de-identified samples were used in the study. Samples were thawed on ice and processed by compressing the Dacron swab to the side of the microfuge tube containing 300 uL of PBS. Protein concentration was measured by spectrophotometric analysis using BCA reagent kit (Thermo Scientific, Rockwell, IL, USA). Clinical samples were analyzed for IL-1β, IL-6, and IL-10 with commercial ELISA assay kits from R & D system (Minneapolis, MN, USA).

Statistical analysis was performed using T-tests for the different viability assays and UPEC growth inhibition assay using Microsoft excel, 2007. Wilcoxon signed-rank tests were used to analyze cytokine data using GraphPad prism 6.

## 4. Conclusions

Urinary tract infections are common among healthy women and are preceded by alterations in the healthy vaginal microbiota, with reduction or loss of the normally protective lactobacilli. We tested for evidence of *in vitro* protective effects mediated by *L*. *crispatus* strain CTV-05, a vaginal probiotic strain that shows promise in restoring the protective vaginal microbiota and preventing recurrent UTI. Using this lactobacillus strain in a system of immortalized vaginal epithelial cells and representative strains of UPEC, we showed: (1) *L*. *crispatus* CTV-05 caused no discernible cell death or damage when incubated with VECs; (2) *L*. *crispatus* CTV-05 protected against cell damage or death caused by UPEC when pre-incubated with VECs prior to exposure to UPEC; (3) when pre-incubated with VECs prior to UPEC exposure, *L*. *crispatus* CTV-05 mitigated some inflammatory cytokine effects; (4) *L*. *crispatus* CTV-05 pre-incubation inhibited the binding of UPEC to VECs; and (5) filtered supernatant from overnight growth of *L*. *crispatus* CTV-05 inhibited UPEC growth. Thus, probiotic *L*. *crispatus* CTV-05 mediates multi-faceted protective mechanisms *in vitro*, from inhibition of UPEC adhesion and proliferation to cytoprotection of VECs from typical uropathogen effects.

## Figures and Tables

**Table 1:** The cytokine response is lower in *L. crispatus* CTV-05 treated vaginal epithelial cells compared to UPEC infected vaginal epithelial cells.

	Control	CTV-05	J96	R45	p^a^	p^b^	p^c^
**IL-1β**	2.49 (0.00–15.7)	4.75 (0.54–11.3)	59.5 (2.85–101)	57.4 (1.89–101)	NS	*	*
**IL-6**	1.16 (0.00–2.56)	2.35 (0.00–4.75)	1.69 (0.41–5.96)	0.95 (0.00–2.78)	NS	NS	NS
**IL-10**	5.25 (0.00–11.3)	9.00 (0.58–51.0)	20.4 (10.9–37.8)	14.2 (8.38–32.8)	NS	NS	NS

IL-1β, IL-6 and IL10 concentrations were determined by ELISA of supernatants from vaginal epithelial cells inoculated with *L. crispatus* CTV-05, *E*. *coli* R45 or *E*. *coli* J96 for 8 h. Values are given in median (min–max). Statistical significance was calculated using Wilcoxon signed-rank test. ns = non-significant, * *p* < 0.05. p^a^ = control *vs. L. crispatus* CTV-05, p^b^ = control *vs. E*. *coli* J96, p^c^ = control *vs. E*. *coli* R45.

**Table 2:** IL-1β secretion is lower in vaginal epithelial cells pre-treated with L. crispatus CTV-05 before UPEC infection compared to only UPEC infected cells.

	R45	R45 + CTV-05	p	J96	J96 + CTV-05	p
**IL-1β**	57.4 (1.89–101)	35.1 (2.13–70.5)	NS	59.5 (2.85–101)	14.7 (2.37–68.8)	*
**IL-6**	0.95 (0.00–2.78)	1.07 (0.00–2.48)	NS	1.69 (0.41–5.96)	1.28 (0.00–3.35)	NS
**IL-10**	14.20 (8.375–32.83)	24.063 (3.38–33.25)	NS	20.3 (10.9–37.8)	23.5 (9.62–64.9)	NS

Supernatant from vaginal epithelial cells pre-treated with *L. crispatus* CTV-05 1 h prior to UPEC infection was used to study the concentrations of IL-1β, IL-6, and IL-10 using ELISAs. IL-1β, IL-6 and IL-10 concentrations were compared to cells infected with only UPEC strains *E*. *coli* R45 or *E*. *coli* J96. Statistical significance was calculated using Wilcoxon signed-rank test. ns = non-significant, * *p* < 0.05.
